# The application of multiplex PCR and PCR-restriction fragment length polymorphism for differentiation of *Bacillus anthracis* from other *Bacillus* spp.

**DOI:** 10.2478/jvetres-2025-0053

**Published:** 2025-10-03

**Authors:** Agnieszka Kędrak-Jabłońska, Sylwia Budniak

**Affiliations:** Department of Bacteriology and Bacterial Animal Diseases, National Veterinary Research Institute, 24-100 Puławy, Poland

**Keywords:** *Bacillus anthracis*, anthrax, *Bacillus cereus* group, PCR, PCR-RFLP

## Abstract

**Introduction:**

*Bacillus anthracis* causes an infectious disease called anthrax. Herbivores are more susceptible to the disease than omnivores, carnivores and humans. Grazing animals are the highest-risk group, and among them, anthrax outbreaks have extremely high fatality rates and impose heavy costs, besides posing a grave zoonotic risk. The aim of the study was the application and evaluation of simultaneous use of multiplex PCR and PCR-restriction fragment length polymorphism (PCR-RFLP) allowing the differentiation of strains of the *B. anthracis* species from other species of the *Bacillus* genus.

**Material and Methods:**

The experiment involved 21 strains of *B. anthracis*. Strains of other species of the *Bacillus* genus were also included in the experiment. In the first part of the studies, two genes responsible for virulence – *pag* and *cap*, located on plasmids pXO1 and pXO2 – and the chromosomal sequence Ba813 were used for a multiplex PCR. In the next stage, PCR-RFLP, in which restriction analysis of the SG-749 sequence using the *Alu*I enzyme was performed.

**Results:**

The multiplex PCR allowed the identification of virulent *B. anthracis* strains, as well as the detection of the presence of the chromosomal sequence Ba813. Then, PCR-RFLP showed the restriction pattern characteristic of *B. anthracis* strains.

**Conclusion:**

The simultaneous use of multiplex PCR and PCR-RFLP enables the distinction of *B. anthracis* strains with and without plasmids from other strains of the *Bacillus* genus, including those with the Ba813 chromosomal sequence.

## Introduction

*Bacillus anthracis* causes an infectious disease called anthrax. Herbivores are particularly susceptible to the disease, which occurs less frequently in omnivores, carnivores and humans. In humans, depending on the route of infection, anthrax can occur in three forms: cutaneous, associated with skin damage and infection; pulmonary, resulting from inhalation of spores; and intestinal, resulting from consumption of contaminated food or water.

The bacterial agent of anthrax is a Gram-positive rod that produces spores in the environment in the presence of oxygen. Vegetative forms are very resistant to low temperatures but are inactivated quickly by higher temperature. In turn, spores are very resistant to unfavourable environmental conditions and can survive in the soil for up to several dozen years (8). Spores kept in soil in the laboratory have been shown to survive for 60 years (29). *B. anthracis* was also isolated from the insulating material of the approximately 110-year-old roof of London’s King’s Cross station (30). Kruger National Park in South Africa has a documented history of periodic anthrax outbreaks (19, 24); studies of approximately 200-year-old animal bones from the park revealed the presence of spores, and growth of *B. anthracis* was achieved from them (28). Cases of anthrax are reported every year around the world in both domestic and wild animals. They have been reported in many countries in Africa, in countries in Asia and the Americas, and in Australia. In the last five years, cases of anthrax have also occurred in Europe, in Albania, Bulgaria, Croatia, France, Germany, Italy, Romania, Russia, Spain and Ukraine (31).

The gold standard for identifying *B. anthracis* is the culture method. Laboratory diagnostic methods of this bacteria based on the assessment of colony morphology and physiological and biochemical properties is time-consuming and requires specialised tests. The classification of bacteria according to phenotypic characteristics is not perfect and does not always give unequivocal results. Genotypic characterisation is more universal than conventional phenotypic methods and facilitates identification and faster detection of the microorganism, determination of its taxonomic position and the study of genetic relationships within the species (26, 30).

The basic molecular biology methods used in the identification of microorganisms are PCR and real-time PCR (4, 20). In the case of *B. anthracis*, they enable the detection of genes located in the pXO1 plasmid, *i.e*. the *pag* gene encoding the protective antigen, the *lef* gene encoding the lethal factor and the *cya* gene encoding the oedema factor. A second plasmid pXO2 contains the *cap* gene sequences (A, B and C), which are responsible for the production of the capsule. However, because the pathogenicity of this microorganism may originate not only in the plasmids but also in the chromosomal sequences, primers for the Ba813 or *rpoB* specific chromosomal markers are also used in addition to primers specific for plasmid DNA sequences (1, 3, 11). PCR and real-time PCR are effective and inexpensive methods for identifying virulent *B. anthracis* strains. However, the presence of avirulent strains that lack plasmids, as well as the occurrence of closely related bacteria of the *Bacillus* genus may cause difficulties in identification. In such cases it becomes necessary to use additional methods to differentiate *B. anthracis* strains. A simple and effective method for identifying and assessing the diversity of bacterial strains, including *B. anthracis*, is PCR-restriction fragment length polymorphism (PCR-RFLP). The basis of this method is the amplification of a specific DNA fragment, which is then digested with appropriately selected restriction enzymes. Electrophoretic analysis of the number and size of the obtained fragments allows the determination of restriction patterns characteristic of a given species, genus or family. This method is characterised by high sensitivity and high specificity. It is also important that it does not require advanced equipment and the costs are not too high (7, 23). The aim of the study was the application and evaluation of simultaneous use of multiplex PCR and PCR-RFLP allowing the differentiation of strains of the *B. anthracis* species from other species of the *Bacillus* genus.

## Material and Methods

### Bacterial strains

The study involved 21 *B. anthracis* strains, including 4 vaccine strains (B.a.v1–v4) and 17 strains isolated from animals between 1947 and 1996 in Poland (B.a.1–3/47, B.a.4/48, B.a.5 and 6/50, B.a.7/51, B.a.8/52, B.a.9–11/53, B.a.12/54, B.a.13–15/93 and B.a.16 and 17/96). Other species of the *Bacillus* genus were also included in the experiment, namely, 13 strains of *B. cereus* (B.c.1–13), 3 strains each of *B. megaterium* (B.m.1–3) and *B. subtilis* (B.s.1–3) and a single strains of *B. mycoides* (B.ms.1) and *B. thuringiensis* (B.t.1). All strains originated from the collection of the Department of Bacteriology and Bacterial Animal Diseases, National Veterinary Research Institute in Puławy, Poland.

### Isolation of DNA

Each strain was streaked on tryptic soy broth (bioMérieux, Marcy-l’Étoile, France) and incubated for 18 h at 37°C. Isolation of DNA was performed using a DNA Genomic Mini Kit (A&A Biotechnology, Gdańsk, Poland). The samples were initially incubated with mutanolysin. The concentration of the obtained DNA was determined spectrophotometrically using a DS-11 spectrophotometer (DeNovix, Wilmington, DE, USA).

### Multiplex PCR

Sequences of primers for amplification were chosen based on the literature data (2, 10, 17). The primers were synthesised in the Laboratory of DNA Sequencing and Synthesis at the Institute of Biochemistry and Biophysics of the Polish Academy of Sciences, Warsaw, Poland. The characteristics of the PCR primers are shown in Table 1.

**Table 1.** The characteristics of the multiplex PCR primers

Target	Primer	Sequence (5′–3′)	Product size	Concentration
*pag*	PA5	TCCTAACACTAACGAAGTCG	596 bp	1.0 μM
	PA8	GAGGTAGAAGGATATACGGT		
*cap*	1234	CTGAGCCATTAATCGATATG	846 bp	0.2 μM
	1301	TCCCACTTACGTAATCTGAG		
Ba813	R1	TTAATTCACTTGCAACTGATGGG	152 bp	0.5 μM
	R2	AACGATAGCTCCTACATTTGGAG		

Multiplex PCRs were performed in a 25 μL reaction mixture containing 100 ng of the DNA, 200 μM of each dNTP (Thermo Fisher Scientific, Carlsbad, CA, USA), 1× PCR buffer, and 1 U of DNA polymerase (Biotools, Madrid, Spain). In order to optimise the reaction, primers with concentrations of 0.2 μM, 0.5 μM, 1.0 μM and 1.5 μM were used. The assay was performed in a TProfessional Basic Thermocycler (Biometra, Jena, Germany) using the following parameters: initial denaturation at 94°C for 5 min; 30 cycles of 94°C for 60 s, 58°C for 90 s and 72°C for 90 s; and a final elongation step at 72°C for 8 min. Electrophoretic separation of the amplification products was performed on a 2% agarose gel in TBE buffer at a constant 95 V in the Wide Mini-Sub Cell GT system (Bio-Rad, Hercules, CA, USA). A 100 bp DNA Ladder Plus (Thermo Fisher Scientific, Vilnus, Lithuania) was used as a molecular weight marker. Gels were stained using SimplySafe (EURx, Gdańsk, Poland). After electrophoresis, gels were photographed using a Vilber Lourmat (Eberhardzell, Germany) imaging system.

### PCR-RFLP

Sequences of primers for amplification were based on literature data in accordance with Daffonchio *et al*. (7). The characteristics of primers are shown in Table 2.

**Table 2.** The characteristics of the PCR-RFLP primers

Target	Primer	Sequence (5′–3′)	Product size	Concentration
SG-749	S-749f	ACTGGCTAATTATGTAATG	749 bp	1.5 μM
	S-749r	ATAATTATCCATTGATTTCG		

The amplification of the SG-749 sequence was performed in a 25 μL reaction mixture containing 100 ng of the DNA, 200 μM of each dNTP (Thermo Fisher Scientific, Carlsbad, CA, USA), 1× PCR buffer, and 1.25 U of DNA polymerase (Biotools, Madrid, Spain). In order to optimise the reaction, primers with concentrations of 0.7 μM, 1.0 μM, 1.5 μM and 2.0 μM were used. This assay was performed in the TProfessional Basic Thermocycler (Biometra, Jena, Germany), using the following parameters: initial denaturation at 94°C for 10 min; 38 cycles of 94°C for 60 s, 50°C for 60 s and 72°C for 120 s; and a final elongation step at 72°C for 5 min. Electrophoretic separation of the amplification products was performed on a 2% agarose gel. A 100 bp DNA Ladder (Thermo Fisher Scientific, Vilnus, Lithuania) was used as a molecular weight marker. The restriction analysis of the SG-749 sequence was conducted using the FastDigest *Alu*I enzyme (Thermo Fisher Scientific, Vilnus, Lithuania). To 5 μL of amplified product, 1 μL of 10× FastDigest Buffer, 0.5 μL of FastDigest *Alu*I and 8.5 μL of water were added. Digestion was performed at 37°C for 15 min, 30 min, 1 h, 2 h, 3 h and 16 h to optimise the reaction. Electrophoretic separation of the products was performed on a 3% agarose gel.

## Results

The multiplex PCR primers enabled the detection of *B. anthracis* genes located in the DNA of plasmids pXO1 and pXO2 and of the Ba813 specific chromosomal sequence (2, 10, 17). For the *pag* gene encoding the protective antigen and located in the DNA of plasmid pXO1, a 596 bp fragment was obtained, and for the *cap* gene encoding the capsule and located in plasmid pXO2, a 846 bp fragment was obtained. The chromosomal sequence yielded a product of 152 bp. Optimisation of multiplex PCR parameters was performed. Table 1 shows the optimal primer concentrations that gave the best visibility of the reaction products.

**Fig. 1.**
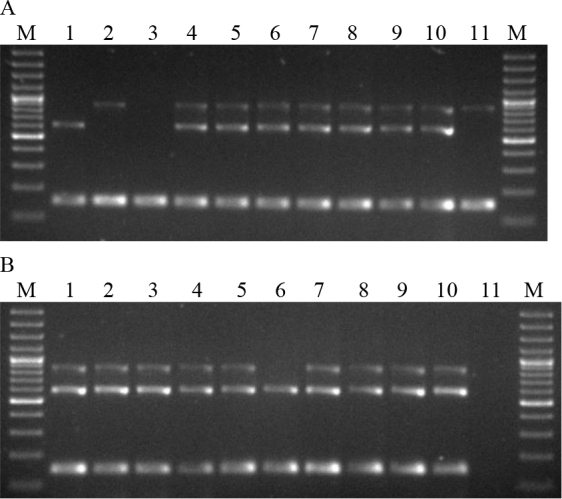
Electrophoresis of multiplex PCR products. A. Lanes: 1–4 B.a.v1–4; 5–7 B.a.1–3/47; 8 B.a.4/48; 9 and 10 B.a.5–6/50; 11 B.a.7/51. B. Lanes: 1 B.a.8/52; 2–4 B.a.9–11/53; 5 B.a.12/54; 6–8 B.a.13–15/93; 9 and 10 B.a.16 and 17/96; 11 negative control; M – molecular weight standard

Figure 1 shows the electrophoresis of multiplex PCR products of the tested *B. anthracis* strains and isolates of other species of the *Bacillus* genus.

**Fig. 2.**
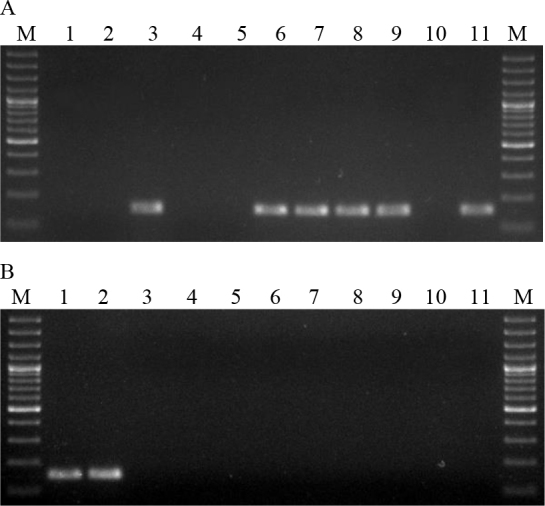
Electrophoresis of multiplex PCR products. A. Lanes: 1–11 B.c.1–11; B. Lanes: 1 and 2 B.c.12 and 13; 3 B.t.1; 4–6 B.m. 1–3; 7 B.ms.1; 8–10 B.s.1–3; 11 negative control; M – molecular weight standard

The B.a.v1 strain contained a 596 bp fragment, indicating the presence of the *pag* gene in the absence of the capsule. In contrast, strain B.a.v2 produced an 846 bp product, indicating the presence of the capsule in the absence of the *pag* gene. Strain B.a.v3 yielded no reaction products, indicating the absence of both the *pag* and *cap* genes. Strains B.a.v4 and 15 of *B. anthracis* originating from animals had both amplified fragments typical of the *pag* and *cap* genes. In two strains isolated from animals, B.a.7/51 and B.a.13/93, the presence of a fragment of 846 bp and 596 bp was detected, respectively. All *B. anthracis* strains showed a 152 bp product indicating the presence of the Ba813 chromosomal sequence. Isolates of other species of the *Bacillus* genus did not show the presence of reaction products of sizes indicating the presence of the *pag* gene and the *cap* gene (Fig. 2). The presence of the Ba813 chromosomal sequence was detected in eight *B. cereus* strains (B.c.3, B.c.6–9 and B.c.11–13). Next, the PCR-RFLP was developed. The optimisation of reaction parameters showed that the concentration of primers for the optimal amplification of the SG-749 sequence was 1.5 μM. This reaction used the SG-749f primer and SG-749r primer to amplify the SG-749 sequence of 4 *B. anthracis* vaccine strains, 17 *B. anthracis* strains isolated from animals and 21 strains of other species of the *Bacillus* genus (Table 3). The SG-749 amplicon of 749 bp was present in all *B. anthracis, B. cereus, B. thuringiensis, B. megaterium* and *B. mycoides* strains tested. However, in *B. subtilis* strains the presence of this sequence was not detected.

**Table 3.** Results of PCR-RFLP

Strain	Presence of the SG-749 sequence	Restriction pattern
*B. anthracis* B.a.v1	+	A
*B. anthracis* B.a.v2	+	A
*B. anthracis* B.a.v3	+	A
*B. anthracis* B.a.v4	+	A
*B. anthracis* B.a.1/47	+	A
*B. anthracis* B.a.2/47	+	A
*B. anthracis* B.a.3/47	+	A
*B. anthracis* B.a.4/48	+	A
*B. anthracis* B.a.5/50	+	A
*B. anthracis* B.a.6/50	+	A
*B. anthracis* B.a.7/51	+	A
*B. anthracis* B.a.8/52	+	A
*B. anthracis* B.a.9/53	+	A
*B. anthracis* B.a.10/53	+	A
*B. anthracis* B.a.11/53	+	A
*B. anthracis* B.a.12/54	+	A
*B. anthracis* B.a.13/93	+	A
*B. anthracis* B.a.14/93	+	A
*B. anthracis* B.a.15/93	+	A
*B. anthracis* B.a.16/96	+	A
*B. anthracis* B.a.17/96	+	A
*B. cereus* B.c.1	+	B
*B. cereus* B.c.2	+	B
*B. cereus* B.c.3	+	C
*B. cereus* B.c.4	+	B
*B. cereus* B.c.5	+	B
*B. cereus* B.c.6	+	C
*B. cereus* B.c.7	+	C
*B. cereus* B.c.8	+	C
*B. cereus* B.c.9	+	C
*B. cereus* B.c.10	+	C
*B. cereus* B.c.11	+	C
*B. cereus* B.c.12	+	D
*B. cereus* B.c.13	+	C
*B. thuringiensis* B.t.1	+	C
*B. megaterium* B.m.1	+	C
*B. megaterium* B.m.2	+	C
*B. megaterium* B.m.3	+	C
*B. mycoides* B.ms.1	+	C
*B. subtilis* B.s.1	–	ND*
*B. subtilis* B.s.2	–	ND
*B. subtilis* B.s.3	–	ND

* ND – not determined

**Fig. 3.**
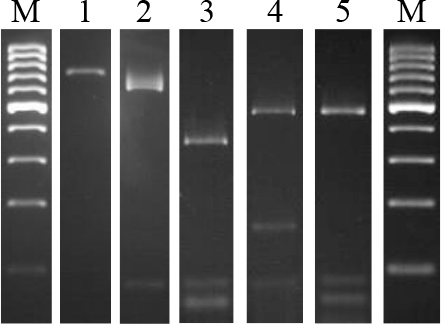
Electrophoresis of PCR-RFLP products. Lanes: 1 SG-749; 2–5 restriction patterns A, B, C and D; M – molecular weight standard

The SG-749 fragment restriction analysis was subsequently optimised using the FastDigest *Alu*I enzyme. The most visible products were obtained with enzyme digestion times of 3 and 16 h, and a digestion time of 3 h was used for further studies. Restriction analysis of the SG-749 sequence in the 4 *B. anthracis* vaccine strains and 17 animal-origin *B. anthracis* strains revealed the presence of two fragments of approximately 90 bp and 660 bp. All *B. anthracis* strains were thus observed to have the same profile. However, as seen in Table 3, restriction analysis of other *Bacillus* spp. revealed their polymorphism. Three species-independent restriction profiles of the *Bacillus* genus were obtained. The obtained restriction profiles differed from the specific profile obtained for *B. anthracis* strains (Fig. 3).

## Discussion

The identification of *B. anthracis* is complicated by this species showing great phenotypic and genotypic similarity to other species of the *Bacillus cereus* group, which also includes *B. cereus, B. thuringiensis, B. mycoides, B. pseudomycoides, B. weihenstephanensis, B. cytotoxicus, B. toyonensis* and *B. wiedmannii* (5, 13, 22, 25). Species of the *Bacillus cereus* group have similar cellular structure and physiology but differ in pathogenicity. Culture and identification of *B. anthracis* from clinical material, and especially from environmental samples, is difficult and time-consuming. In the case of environmental samples, the difficulty is related to the presence of other aerobic spore-forming bacteria as well as other bacteria of the *Bacillus* genus. Identification is based on phenotypic differences between *B. anthracis* and other species in the genus, *i.e*. colony morphology, haemolysis, lack of motility, ability to produce a capsule and susceptibility to penicillin and gamma phage (28). In addition to typical *B. anthracis* strains, strains with atypical characteristics may be present; therefore, it is not always possible to distinguish *B. anthracis* from other *Bacillus* species using conventional bacteriological methods. Penicillin-resistant strains are such atypical *B. anthracis* (30). Hugh-Jones *et al*. (9) found that some *B. anthracis* strains may be resistant to bacteriophage gamma, whereas *B. cereus* strains were reported to be susceptible. Also, as reported by Logan *et al*. (14), the assessment of biochemical features cannot unequivocally determine the species affiliation of a strain to the *Bacillus* genus.

Closely related bacteria from the *Bacillus* genus may also cause inconclusive results in the molecular diagnosis of *B. anthracis* because of their genomic similarity to this species (26). As shown by Kuske *et al*. (12) and Marston *et al*. (15), this is particularly a problem when analysing environmental samples, and molecular studies must be detailed for accurate and conclusive *B. anthracis* identification. With the progress in molecular biology methods, sensitive and specific PCR and real-time PCR tests have been developed and are used to identify *B. anthracis*. In our studies, multiplex PCRs with specific primers for detection of the *B. anthracis pag* gene located on the DNA of plasmid pXO1, the *cap* gene located on the DNA of plasmid pXO2 and the Ba813 chromosomal marker were used (1, 11). Sixteen *B. anthracis* strains, including one vaccine strain, harboured plasmids pXO1 and pXO2. Single strains, both vaccine and originating from animals, lacked one of the plasmids, and one vaccine strain was characterised by the absence of both plasmids. However, the presence of the chromosomal sequence Ba813 was demonstrated in all *B. anthracis* strains, but also in 8 of 13 *B. cereus* strains.

Both plasmids are detected in virulent *B. anthracis* strains. However, studies by Cooper *et al*. (6), Marston *et al*. (16), Patra *et al*. (17, 18) and Turnbull (26) indicated the possibility of the occurrence of strains lacking one or both plasmids. Such strains may appear especially in environmental samples that are exposed to various physical and chemical factors leading to changes in the bacterial genome and plasmids. Turnbull (26) indicated the possibility of natural loss of one or both plasmids caused by unidentified environmental stresses. He also found a more frequent occurrence of strains lacking the pXO2 plasmid and occasional strains lacking both plasmids. In their studies, Patra *et al*. (17) confirmed the occurrence of *B. anthracis* strains lacking both plasmids among bacteria isolated from the environment. These researchers also showed that in the case of strains originating from animals, strains without the pXO2 plasmid appeared. In turn, Marston *et al*. (16) found that long-term storage in the laboratory may cause loss of pXO1 or pXO2 or both plasmids. Thus, the presence of avirulent strains that lack the genes responsible for toxin production and capsule formation occurs primarily in the environment.

In their studies, Patra *et al*. (17) found the chromosomal marker Ba813 useful for identifying *B. anthracis* strains lacking plasmids. However, in a later article, they showed that the Ba813 sequence was present in some strains belonging to other species of the *Bacillus* genus (18). Ramisse *et al*. (21) also demonstrated the occurrence of the Ba813 sequence in one strain of *B. thuringiensis* and three strains of *B. cereus*. This was confirmed by the results of our own research, as we found Ba813 in several *B. cereus* strains.

The difficulties in identifying *B. anthracis* strains and distinguishing them from other closely related strains of the *Bacillus cereus* group have prompted researchers to look for other specific chromosomal markers and methods. Daffonchio *et al*. (7) used the PCR-RFLP technique, which made possible the distinction of *B. anthracis* strains from other species belonging to the *Bacillus* genus. This method allowed the detection of the SG-749 sequence present in strains belonging to the *Bacillus cereus* group. The researchers found SG-749 in all tested *B. anthracis* strains, as well as in *B. cereus, B. thuringiensis* and *B. mycoides* strains. In our own work, we also confirmed the occurrence of this sequence in *B. anthracis* possessing and lacking one or both plasmids, as well as in *B. cereus, B. thuringiensis, B. megaterium* and *B. mycoides*.

In order to distinguish *B. anthracis* strains from other closely related species of the *Bacillus* genus, restriction analysis using the selected enzyme was performed after amplification of the SG-749 fragment. Using the *Alu*I enzyme, Daffonchio *et al*. (7) obtained 11 restriction groups in the tested strains of the *Bacillus* genus. All *B. anthracis* strains resolved to the same profile, in which they obtained two restriction fragments characteristic of this species, approximately 90 and 660 bp in size. However, they found 10 species-independent restriction profiles in other strains of the *Bacillus* genus, demonstrating high sequence diversity. In our own research, one common restriction pattern was also found in both *B. anthracis* strains with and without plasmids. However, similarly to the studies by Daffonchio *et al*. (7), the tested strains of *B. cereus, B. thuringiensis, B. megaterium* and *B. mycoides* were characterised by high species-independent polymorphism. Thus, the restriction profile was characteristic of *B. anthracis* and allowed distinguishing strains of this species from other strains of the *Bacillus* genus.

In summary, the simultaneous use of multiplex PCR and PCR-RFLP allows the rapid and accurate discrimination of the strains of the species *B. anthracis*, with and without plasmids, from other species in the genus.

## Conclusion

By detecting both virulence markers, multiplex PCR grant the possibility of identifying virulent *B. anthracis* strains as well as detecting the Ba813 chromosomal sequence. In turn, PCR-RFLP enables the distinction of strains of this species from other strains of the *Bacillus* genus, including those with the Ba813 chromosomal sequence.
